# Anger and aggression in borderline personality disorder and attention deficit hyperactivity disorder – does stress matter?

**DOI:** 10.1186/s40479-017-0057-5

**Published:** 2017-03-17

**Authors:** Sylvia Cackowski, Annegret Krause-Utz, Julia Van Eijk, Katrin Klohr, Stephanie Daffner, Esther Sobanski, Gabriele Ende

**Affiliations:** 1 Department of Psychosomatic Medicine and Psychotherapy, Central Institute of Mental Health Mannheim, Medical Faculty Mannheim/Heidelberg University, J5, D-68159 Mannheim, Germany; 2 Department of Clinical Psychology, Faculty of Social and Behavioural Science, Leiden University, Leiden, The Netherlands; 3 Department of Neuroimaging, Central Institute of Mental Health, Medical Faculty Mannheim/Heidelberg University, Mannheim, Germany; 4 Department of Psychiatry and Psychotherapy, Central Institute of Mental Health, Medical Faculty Mannheim/Heidelberg University, Mannheim, Germany

**Keywords:** Borderline personality disorder, Attention deficit hyperactivity disorder, Anger, Aggression, Impulsivity, Emotion regulation, Stress

## Abstract

**Background:**

The impact of stress on anger and aggression in Borderline Personality Disorder (BPD) and Attention Deficit Hyperactivity Disorder (ADHD) has not been thoroughly investigated. The goal of this study was to investigate different aspects of anger and aggression in patients with these disorders.

**Methods:**

Twenty-nine unmedicated female BPD patients, 28 ADHD patients and 30 healthy controls (HC) completed self-reports measuring trait anger, aggression and emotion regulation capacities. A modified version of the Point Subtraction Aggression Paradigm and a state anger measurement were applied under resting and stress conditions. Stress was induced by the Mannheim Multicomponent Stress Test (MMST).

**Results:**

Both patient groups scored significantly higher on all self-report measures compared to HCs. Compared to ADHD patients, BPD patients reported higher trait aggression and hostility, a stronger tendency to express anger when provoked and to direct anger inwardly. Furthermore, BPD patients exhibited higher state anger than HCs and ADHD patients under both conditions and showed a stress-dependent anger increase. At the behavioral level, no significant effects were found. In BPD patients, aggression and anger were positively correlated with emotion regulation deficits.

**Conclusions:**

Our findings suggest a significant impact of stress on self-perceived state anger in BPD patients but not on aggressive behavior towards others in females with BPD or ADHD. However, it appears to be pronounced inwardly directed anger which is of clinical importance in BPD patients.

**Electronic supplementary material:**

The online version of this article (doi:10.1186/s40479-017-0057-5) contains supplementary material, which is available to authorized users.

## Background

Affect dysregulation and related problems with impulsivity, anger control deficits and aggression constitute a characterizing symptom cluster in Borderline Personality Disorder (BPD) [1–3] and Attention Deficit Hyperactivity Disorder (ADHD) [4–7]. Aggression in BPD patients manifests itself in self-destructive behavior (e.g., high risk behavior, self-injury) or externally directed (impulsive) aggression [8, 9]. The latter can also be observed in ADHD patients and is reflected in low frustration tolerance and recurrent temper tantrums [5]. Impulsive aggression is characterized by behavioral disinhibition, alongside a lack of planning and concerns about consequences [10].

The occurrence of aggressive behavior may be influenced by different personal or situational variables (for overview see [11]), such as gender [12, 13], educational level, income [14], certain personality traits (e.g. impulsivity) [15, 16] or provocation [17, 18]. Gender differences have been frequently discussed in aggression research and the type of aggression appears to play a crucial role [12, 13]. Evidence has shown that men are physically more aggressive, but not more aggressive in general, and that provocation evokes aggression to the same extent in men and women [13]. Some studies also support these findings in BPD patients [18–20].

There is further indication that unspecific affective arousal or stress can enhance the likelihood of aggressive behavior [11, 21]. This should be taken into account when investigating aggression in BPD, as these patients frequently experience high levels of aversive arousal [22, 23]. While there is evidence for stress effects on the related construct of impulsivity in BPD patients [24–27], previous studies examining aggression in BPD have not systematically investigated the influence of stress. Furthermore, the high comorbidity rates of BPD with substance disorder, bipolar disorder, antisocial personality disorder and ADHD [28–30] are important to consider, as these disorders are already associated with elevated levels of impulsivity and aggression [27, 31–34].

Previous studies, which used well-established self-rating scales (i.e. the State-Trait Anger Expression Inventory, STAXI; [35], Buss-Perry Aggression Questionnaire, BPAQ; [36]), revealed elevated levels of anger and aggression in BPD patients [18–20, 24, 37, 38]. McCloskey et al. [19] found significantly higher scores in trait anger and aggression in female and male BPD patients compared to healthy controls (HCs) and patients with non-cluster-B personality disorders. Beyond self-report measures, the Point Subtraction Aggression Paradigm (PSAP; [39]) has been frequently used for the behavioral assessment of aggression (in terms of point-subtracting responses to a fictitious- opponent), and has already been applied to BPD patients [18–20, 37]. For example, New et al. [18] demonstrated that a gender-mixed sample of BPD patients with intermittent explosive disorder reacted more frequently with aggressive responses in the PSAP compared to HCs. However, in this study, it was unclear whether comorbid intermittent explosive disorder at least partly explained elevated aggression scores in BPD.

Although previous studies excluded comorbid conditions such as bipolar disorder or current substance abuse [18, 19], to our knowledge, no previous studies have controlled for comorbid ADHD. Adult ADHD is a highly prevalent comorbid condition in BPD patients (about 38%; [28, 30, 40]) and is also characterized by impulsivity and anger control problems [4, 5]. Previous research in adult ADHD samples has revealed higher self-reported trait anger and poorer anger control (STAXI; [35]) in ADHD patients compared to HCs [32, 41] and also compared to a control group with low ADHD symptoms [42]. In the latter study, individuals in the ADHD group reported significantly higher anger, lower anger control and more dysfunctional anger expression (e.g., noisy arguing, physical aggression directed towards objects). Studies with self-report measures of anger and aggression comparing ADHD and BPD patients are scarce and provide partly inconsistent finding [32, 41]. Although there are many studies indicating an impaired behavioral inhibition in ADHD patients compared to HCs [27, 32, 43, 44], little is known regarding aggression in adult ADHD patients. Most studies assessing aggressive behavior have been conducted with children and adolescents [45–47], but studies examining aggression in adult ADHD patients (especially females) compared to healthy and clinical control groups are lacking.

The aim of this study was to further investigate the nature of anger and aggression in BPD and ADHD patients by examining the impact of stress on these features, while controlling for comorbid ADHD in BPD patients and vice versa. In the main study, we hypothesized that female BPD and ADHD patients would show higher scores in self-report measures of anger and aggression compared to healthy women. We were also interested in a potential group difference and stress condition effect in self-reported state anger and behavioral aggression. We expected more state anger and aggressive responses in patients after stress induction compared to HCs. Another aim of our study was to investigate correlations between self-reported emotion regulation capacities and measures of aggression in female BPD and ADHD patients.

## Methods

### Sample

A total number of 93 females between 18 and 43 years of age participated in the study. Recruitment took place at the Department of Psychosomatic Medicine and Psychotherapy and the Department of Psychiatry and Psychotherapy at the CIMH. Participants were additionally recruited via advertisements in newspapers, on websites and on disorder-specific internet forums, as well as through flyers for therapists. The BPD and ADHD sample consisted of outpatients and patients who currently did not make use of psychotherapeutic treatment. None of the participants was in inpatient treatment as the investigation took place.

Five participants had to be excluded from final data analysis in the main study: two HCs were excluded due to drug abuse and a diagnosis of current dysthymia, one BPD patient cancelled participation before study completion and data of two other BPD patients could not be obtained due to technical difficulties. The final sample consisted of 29 female patients with BPD, 28 with ADHD and 30 HCs.

### Clinical diagnostics and basic assessments

All participants underwent diagnostic assessments including the Structured Clinical Interview for DSM-IV Axis-I (SCID-I; [48]) and the Borderline Section of the International Personality Disorder Examination (IPDE; [49]; inter-rater-reliability κ =0.77). In addition, the Standard Progressive Matrices Test (SPM; [50]) was completed by all participants in order to estimate intelligence.

Further clinical variables were assessed with questionnaires for borderline symptom severity (Borderline Symptom List-23, BSL-23; [51]) and dysphoric mood (Beck Depression Inventory II, BDI-II; [52]). The Barratt Impulsiveness Scale-11 (BIS-11; [53]) was applied as a measure for impulsivity. Emotion regulation capacities were assessed by the Difficulties in Emotion Regulation Scale (DERS; [54]). A higher DERS total score implies better emotion regulation capacities. Subjective stress levels during the experiment were rated on a ten-point Likert scale (0 = “not at all” to 9 = “extremely”).

### Inclusion and exclusion criteria

For inclusion into the BPD group patients had to fulfil at least five DSM-IV criteria for BPD [53] as assessed by the IPDE. For verification of ADHD diagnosis, as well as exclusion of ADHD diagnosis in BPD patients, four different measurements (validated German versions) were applied: 1) The short version of the Wender Utah Rating Scale (WURS-k; [55]) was used to assess childhood ADHD symptoms. This self-report scale consists of 25 items which are answered on a five-point Likert scale (0 = “not applicable” to 4 = “applicable”). For the assessment of ADHD symptoms in adulthood 2) the ADHD-Self-Rating scale (ADHD-RS; [56]) and 3) the Connor Adult ADHD Rating Scale - Self-Report: Long Version (CAARS-S:L; [57]) were used. Both scales are based on the DSM-IV criteria for ADHD [58]. Furthermore, 4) the Wender-Reimherr Adult Attention Deficit Disorder Scale (WRAADDS; [59]) was applied, which is a clinical interview conceptualized for adult ADHD to assess the core features of inattention, hyperactivity and impulsivity, and additional features comprising temperament, affective lability, stress tolerance and disorganization. Experienced clinical psychologists and psychiatrists clarified possible inconsistencies in the self-measurements by the WRAADDS [59] and integrated external assessments (e.g. school reports, interviews with parents or relatives) to reach the diagnosis of ADHD. Only in case of clear verification of the ADHD symptomatology patients were included in the study. In ADHD patients, a possible BPD diagnosis was excluded via the IPDE.

Exclusion criteria for all participants comprised the use of psychotropic medication within two weeks prior to study, significant somatic disorders, pregnancy or mental deficiency. A few patients (11%) gradually reduced intake of their psychotropic medication and stopped intake two weeks before the study took place. Approval for this procedure was given only, if certain conditions were met: medication was reduced in consultation with the attending physician of the patient, the general state and living conditions were mostly stable and the patient had the intention to try a medication free period anyway. Lifetime history of any psychiatric disorder was an exclusion criterion for HCs. BPD and ADHD patients were excluded if they had a lifetime history of bipolar affective disorder or psychotic disorder, a current suicidal crisis and/or substance abuse within the last two months (a lifetime diagnosis of substance dependence was allowed). All clinical assessments and interviews were conducted by well-trained clinical psychologists and psychiatrists.

### Self-report measures of anger and aggression

Subjects completed three questionnaires assessing anger and aggression: the Brown-Goodwin Lifetime History of Aggression (BGLHA; [60]), the Buss-Perry Aggression Questionnaire (BPAQ; [36]) and the State-Trait Anger Expression Inventory (STAXI; [35]). The BGLHA assesses instances of fighting, assaults, temper tantrums, school discipline problems, problems with superiors, antisocial behavior not involving police, as well as antisocial behavior involving police. Each item is rated on a scale from 0 to 4, indicating the frequency of antisocial events ranging from “never” to “more than four times”. The BPAQ is a measure of trait aggressiveness with 29 items related to four subscales: anger, hostility, physical and verbal aggressiveness. Participants rate the extent to which each item characterizes themselves from 1 (extremely uncharacteristic) to 4 (extremely characteristic). The trait part of the STAXI assesses one’s disposition to experience anger and consists of the two subscales “temperament” (propensity to experience anger without specific provocation) and “reaction” (anger experience when provoked). Anger expression is gathered via three subscales: “anger in” (tendency to suppress angry feelings), “anger out” (tendency to express anger toward other people or objects) and “anger control” (ability to control expressions of anger). The state of the STAXI was developed for repeated measurement and measures the intensity of current subjective anger. All items are rated on a 4-point Likert scale ranging from 1 (not at all/almost never) to 4 (very much/almost always).

### Behavioral assessment of aggression

The Point Subtraction Aggression Paradigm (PSAP; [39]) is a widely used computer-based measure of aggressive responses to provocation. The participant is instructed to accumulate points, which can be exchanged for money. Provocation through point subtractions during the game is ascribed to another player but is in fact pre-determined by the program. Three different action options (buttons) are given: 1) by pressing button A approximately 100 times, ten points are earned; 2) by pressing button B ten times, ten points from the (fictitious) opponent are subtracted; and 3) by pressing button C ten times, the participant can protect his points from point subtractions by the opponent. After the B or C button is pressed, a provocation free interval (PFI) is started, during which no point subtraction occurs. The number of button B responses is used as an indicator of aggression, as B button presses deliver an aversive stimulus through point-subtraction to the opponent.

There exist several versions of the PSAP, which differ for example in the number of buttons (two buttons vs. three buttons) (i.e. [61, 62]), number and duration of sessions (10 minutes or 25 minutes, repeated twice or more) (i.e. [63–65]) or the PFIs (45 seconds – 500 seconds) (i.e. [37, 64, 65]).

In the present study, a 12.5-minute version of the PSAP with a high provocation-rate was used (provocations occurred every 6–60 seconds and PFI was set at 31.25 seconds) in order to adapt the PSAP to our test battery (which also comprised other laboratory tasks; see [27]). In our study, a video recording of the opponent was shown during the whole session in the top right corner of the computer screen, and the participant was told that the opponent would also see the participant via a webcam. Because of the modifications, a pilot study with male BPD and ADHD patients and HCs was conducted to test whether our version of the PSAP was sensitive for stress-dependent changes in behavioral aggression (see Additional file 1).

### Stress induction

For stress induction, the Mannheim Multicomponent Stress Test (MMST; [66, 67]) was used, which consists of a combination of an emotional (aversive pictures), a sensory (white noise displayed over headset), a cognitive (calculation under time pressure: Paced Auditory Serial Addition Task (PASAT-C); [68]), and a motivational (loss of money due to calculation errors) stressor. To ensure that the stress induction was successful, subjective stress was assessed with a 10-point Likert scale, as well as via heart rate.

### Procedure

This study was approved by the Ethics committee of the Medical Faculty Mannheim/Heidelberg University and was conducted in accordance with the Declaration of Helsinki. After participants were informed about the background and procedure of the experiment, written informed consent was obtained and participants underwent diagnostics and completed the basic clinical assessments.

Participants completed the PSAP on two different days (within a 3-day interval). The order of the resting and stress conditions was randomized. During both sessions, participants completed the STAXI state part and the Likert scale for subjective stress. The STAXI was completed before and after PSAP performance (analyses were performed with the means of the two scores). Additionally, at the stress session a baseline heart rate assessment was conducted for five minutes. Afterwards, the stress induction with the MMST was conducted for five minutes, while heart rate was measured simultaneously. Heart rate was assessed in five seconds intervals by a chest belt and wirelessly transmitted to the heart rate receiver attached to the participant’s wrist. Subsequently the subjective stress rating and the STAXI state were completed and the PSAP was started. At the end of the study, participants were debriefed, thanked and paid for their participation.

### Data analysis

The congruence of data with normal distribution assumptions was tested using Kolmogorov-Smirnov tests. Some scores in the main study were found to be not congruent with normal distribution. Therefore, differences between groups were initially tested using nonparametric tests (Mann–Whitney *U*, Kruskal-Wallis *H* and Wilcoxon test). Because there were no differences in the patterns of results when using nonparametric tests versus parametric tests (analyses of variance (ANOVA) or multivariate ANOVA (MANOVA) and students’ t-tests), the results of parametric analyses are presented for the purpose of simplicity. State variables (anger, aggression, stress ratings, heart rate) were analyzed using 3× 2 repeated measure ANOVAs (rm-ANOVA) with group (HC vs. BPD vs. ADHD) as between-factor and condition (resting vs. stress) as within-factor. In case of significant effects, post-hoc Tukey-HSD tests were used for group comparisons and paired t-tests for within-group comparisons. Bivariate Pearson’s product–moment correlations between self-reported emotion regulation capacities (DERS) and the total scores of the anger and aggression measures were computed in BPD and ADHD patients. Bonferroni correction was used to account for multiple comparisons. Threshold for statistical significance was set at *p* < 0.05, two-tailed. Effect sizes partial eta squared (η_p_^2^), Cohen’s *d* [69] and Cramér’s V (φ_c_) are reported in case of significant effects.

## Results

### Demographic and clinical variables

The means and SD for demographic and clinical variables, as well as patients’ comorbid psychiatric disorders are presented in Table 1.

**Table 1:** Demographic and clinical variables in healthy control participants (HC), patients with Borderline Personality Disorder (BPD) and patients with Attention Deficit Hyperactivity Disorder (ADHD)

	HC (*n*= 30) M ± S.D.	BPD (*n*= 29) M ± S.D.	ADHD (*n*= 28) M ± S.D.	*F/χ* ^*2*^	*p*	*η*_*p*_^*2*^ */φ*_*c*_
Age	27.53 ± 6.60	27.07 ± 6.51	30.11 ± 7.96	1.70	.189	
Intelligence (IQ)						
Raven SPM	111.70 ± 10.50	107.07 ± 12.32	105.46 ± 12.71	2.18	.119	
Income, *n (%)*						
100-300€	7 (23)	8 (28)	6 (21)			
350-500€	6 (20)	3 (10)	4 (14)	3.00	.934	
550-700€	7 (23)	6 (21)	4 (14)			
750-1000€	3 (10)	3 (10)	3 (11)			
+ 1000€	7 (23)	9 (31)	11 (39)			
Years of education, *n (%)*						
Less than 9 years	0 (0)	0 (0)	4 (14)			
9 years	0 (0)	1 (3)	3 (11)	15.60	.016^b^	.30
10 years	8 (27)	9 (31)	10 (36)			
13 years	22 (73)	19 (66)	11 (39)			
Current co-morbidities, *n (%)*						
MDD		4 (14)	1 (4)			
Anxiety disorder		11 (38)	5 (18)			
Substance abuse		0 (0)	0 (0)			
Eating disorder		8 (28)	3 (11)			
PTSD		13 (45)	1 (4)			
OCD		3 (10)	0 (0)			
Medication, *n (%)* ^*d*^						
No stable medication	30 (100)	23 (88)	25 (89)			
Intake stopped 2 week prior to the study	0 (0)	3 (12)	3 (11)			
WURS-k	6.67 ± 5.71	29.62 ± 16.40	40.29 ± 13.83	53.11	≤.001^a,b,c^	.56
ADHD-RS	6.53 ± 5.45	19.93 ± 8.68	34.25 ± 7.65	102.64	≤.001^a,b,c^	.71
CAARS	31.10 ± 17.59	89.66 ± 26.10	121.18 ± 23.58	118.91	≤.001^a,b,c^	.74
BSL23	2.60 ± 4.12	47.90 ± 20.54	17.18 ± 11.89	81.74	≤.001^a,b,c^	.66
BDI-II	2.17 ± 3.26	32.00 ± 11.55	16.46 ± 11.35	73.30	≤.001^a,b,c^	.64
BIS-11	53.40 ± 7.43	66.45 ± 10.49	81.29 ± 10.42	62.16	≤.001^a,b,c^	.60
DERS	145.00 ± 12.92	85.14 ± 19.31	103.18 ± 19.59	91.31	≤.001^a,b,c^	.69

Note: Data are presented in means ± standard deviations, statistical group comparisons by analysis of variance (degrees of freedom (*df*): *F*_(2, 84)_) and *χ* ^*2*^ -test for income (*df* = 8) and education (*df* = 6); *p*-value; effect size in *η*_*p*_^*2*^ and φ_c_;

*MDD* Major Depressive Disorder, *PTSD* Posttraumatic stress disorder, *OCD* Obsessive Compulsive Disorder, *WURS-k* Wender Utah Rating scale short version, *ADHD-RS* Attention Deficit Hyperactivity self-rating scale, *CAARS* Connor Adult ADHD Rating Scale, *BSL-23* Borderline Symptom List-23, *BDI-II* Beck Depression Inventory II, *BIS-11* Barratt Impulsiveness Scale, *DERS* Difficulties in Emotion Regulation Scale

^a^HC vs. BPD significant differences

^b^HC vs. ADHD significant differences

^c^BPD vs. ADHD significant differences

^d^The mentioned percentages refer to 26 of the 29 patients with BPD. For the remaining 3 BPD patients information regarding the medication (whether medication-free or intake was stopped 2 week prior to the study) was either missing or not entirely conclusive retrospectively.

There were no significant differences in demographic variables, except for the education level, with ADHD patients showing fewer years of education than HCs. All three groups differed significantly in the BDI, the BIS-11 and the DERS. While BPD patients showed the highest BDI scores and the lowest DERS score, the most elevated BIS-11 scores were found in ADHD patients. As expected, BPD patients reported significantly higher BSL23 scores than HCs and ADHD patients. For further characterization of the samples, also the ADHD scales were listed in Table 1. In all ADHD scales, ADHD patients showed highest scores.

### Manipulation check: Stress induction

Means with SD and statistics for subjective stress ratings and heart rate are depicted in Table 2. The rm-ANOVA with heart rate as dependent variable revealed a significant main effect of Condition (*F*_(1,82)_ = 134.81, *p* ≤ .001, *η*_*p*_^*2*^ = 0.62), with significantly increased heart rates after stress induction in all three groups. In the rm-ANOVA for subjective stress, also a significant main effect of Condition was found (*F*_(1,84)_ = 86.51, *p* ≤ .001, *η*_*p*_^*2*^ = 0.51), indicating significantly higher subjective stress in the stress condition. Furthermore, there was a significant main effect of Group (*F*_(1,84)_ = 18.38, *p* ≤ .001, *η*_*p*_^*2*^ = 0.30), with both patient groups reporting higher stress levels than HCs under both conditions, but no significant interaction effect (*F*_(2,84)_ = 1.77, *p* = .177).

**Table 2:** Ratings of subjective stress and heart rate in resting condition and stress condition in healthy controls (HC), patients with Borderline Personality Disorder (BPD) and patients with Attention Deficit Hyperactivity Disorder (ADHD)

	Stress ratings M ± S.D.		Paired *t*-tests	Heart rate M ± S.D.		Paired *t*-tests
	Resting condition	Stress condition		Resting condition	Stress condition	
HC (*n* = 30)	1.63 ± 1.47	4.33 ± 2.20	*t*_(29)_ = −6.50 *p* ≤ .001 *d* = 1.44	79.48 ± 11.43	101.10 ± 19.63	*t*_(29)_ = −6.69 *p* ≤ .001 *d* = 1.35
BPD^a^ (*n* = 29)	3.76 ± 1.70	5.72 ± 2.17	*t*_(28)_ = −4.10 *p* ≤ .001 *d* = 1.01	80.96 ± 10.54	97.74 ± 16.92	*t*_(26)_ = −7.53 *p* ≤ .001 *d* = 1.19
ADHD (*n* = 28)	3.50 ± 2.25	6.79 ± 1.89	*t*_(27)_ = −5.65 *p* ≤ .001 *d* = 1.58	80.53 ± 13.60	93.57 ± 17.07	*t*_(27)_ = −6.89 *p* ≤ .001 *d* = 0.85
ANOVA	*F*_(2, 84)_ = 11.86 *p* ≤ .001^b,c^ *η*_*p*_^*2*^ = 0.22	*F*_(2, 84)_ = 10.00 *p* ≤ .001^b,c^ *η*_*p*_^*2*^ = 0.19		*F*_(2, 82)_ < 1 *p* = .939	*F*_(2, 82)_ = 1.27 *p* = .285	

Note: ^a^ = heart rate data of two BPD patients are missing due to technical problems (*n* = 27)

^b^ HC vs. BPD significant differences

^c^ HC vs. ADHD significant differences

### Self-reported trait anger and aggression

Table 3 presents the means with SD and statistics of the STAXI, BPAQ and BGLHA subscales and total scales, which were completed by the participants once within the frames of the diagnostic procedure. Univariate ANOVAs using the total scores of the STAXI, BPAQ and BGLHA as dependent variables revealed a significant main effect of Group. For every score, post-hoc tests showed significant differences between BPD and HC (all *p* ≤ .001), as well as between ADHD and HC (all *p* ≤ .001), with higher scores in BPD and ADHD patients than in HCs. Compared to ADHD patients, BPD patients also showed significantly higher ratings in the BPAQ total score (*p* = .020). MANOVAs with the STAXI subscales “temperament” and “reaction” (*F*_(4,166)_ = 16.09, *p* ≤ .001, *η*_*p*_^*2*^ = 0.28) and the three expression scales “anger in”, “anger out” and “anger control” (*F*_(6,164)_ = 21.55, *p* ≤ .001, *η*_*p*_^*2*^ = 0.44) also showed significant effects of group. Post-hoc analyses revealed that both BPD and ADHD patients scored higher on the temperament, reaction, anger in and anger out scales and lower on the anger control scale than HCs (all *p* ≤ .001, except HC vs. BPD in STAXI control *p* = .002). Group differences between BPD and ADHD were also significant in the reaction (*p* = .024) and anger in scale (*p* ≤ .001), with BPD patients reporting higher scores.

**Table 3:** Means and standard deviation of STAXI, BPAQ and BGLHA scores and results of the univariate ANOVAs (*F*-ratio, *p*-value and effect size) in healthy controls (HC), patients with Borderline Personality Disorder (BPD) and patients with Attention Deficit Hyperactivity Disorder (ADHD)

	HC (*n* = 30) M ± S.D.	BPD (*n* = 29) M ± S.D.	ADHD (*n* = 28) M ± S.D.	*F*	*p*	*η*_*p*_^*2*^
Trait measures						
STAXI						
Total ^b,c^	16.23 ± 3.53	29.52 ± 7.26	25.75 ± 6.96	37.00	≤.001	.47
Temperament ^b,c^	7.43 ± 1.85	13.93 ± 4.51	12.43 ± 3.86	26.80	≤.001	.39
Reaction ^b,c,d^	8.80 ± 2.31	15.59 ± 3.38	13.32 ± 3.77	34.55	≤.001	.45
Anger in ^b,c,d^	12.53 ± 3.37	23.72 ± 5.01	18.25 ± 5.36	42.95	≤.001	.51
Anger out ^b,c^	11.30 ± 2.60	18.69 ± 6.22	18.79 ± 4.63	24.59	≤.001	.37
Anger control ^b,c^	23.90 ± 4.40	19.86 ± 5.19	17.32 ± 3.36	16.62	≤.001	.28
BPAQ						
Total ^b,c,d^	44.97 ± 8.74	74.48 ± 14.98	64.93 ± 14.95	38.77	≤.001	.48
Anger ^b,c^	12.30 ± 3.57	19.17 ± 4.37	19.71 ± 4.71	28.12	≤.001	.40
Physical ^b,c^	11.03 ± 2.48	18.55 ± 7.45	15.57 ± 6.25	12.76	≤.001	.23
Verbal ^b,c^	9.57 ± 2.00	12.31 ± 4.23	12.00 ± 3.62	5.78	.004	.12
Hostility ^b,c,d^	12.07 ± 3.86	24.45 ± 5.12	17.64 ± 5.43	48.51	≤.001	.54
BGLHA^a^						
Total ^b,c^	1.23 ± 1.94	10.48 ± 7.23	7.85 ± 4.66	26.01	≤.001	.39

Note: *STAXI* State-Trait Anger Expression Inventory, *BPAQ* Buss Perry Aggression Questionnaire, *BGLHA* Brown-Goodwin Lifetime History of Aggression

^a^BGLHA: smaller sample size due to missing values: BPD (*n* = 27) and ADHD (*n* = 27)

^b^HC vs. BPD significant differences

^c^HC vs. ADHD significant differences

^d^BPD vs. ADHD significant differences

There was a main effect of group in the MANOVAs for the BPAQ subscales anger, hostility, physical and verbal aggression (*F*_(8,162)_ = 16.98, *p* ≤ .001, *η*_*p*_^*2*^ = 0.46). Post-hoc analyses revealed that BPD and ADHD patients both rated themselves significantly higher on all four subscales compared to HCs (all *p* ≤ .001, except HC vs. BPD for verbal aggression: *p* = .007; HC vs. ADHD for verbal aggression: *p* = .021 and physical aggression: *p* = .010). Furthermore, patient groups differed from each other on the hostility subscale, with BPD patients reporting more hostility than ADHD patients (*p* ≤ .001).

### Self-reported state anger

**Fig. 1:**
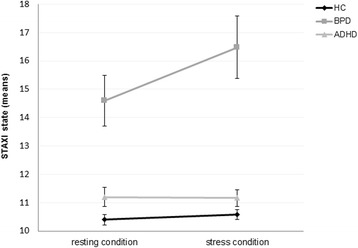
Means with standard errors of self-reported state anger (STAXI) under resting and stress conditions in healthy controls (HC), patients with Borderline Personality Disorder (BPD) and patients with Attention Deficit Hyperactivity Disorder (ADHD)

Figure 1 shows the means with standard errors of STAXI state scores under resting and stress conditions. The rm-ANOVA revealed a significant main effect of condition (*F*_(1,84)_ = 5.49, *p* = .022, *η*_*p*_^*2*^ = 0.06), a main effect of group (*F*_(2,84)_ = 23.72, *p* ≤ .001, *η*_*p*_^*2*^ = 0.36), as well as a significant condition x group interaction effect (*F*_(2,84)_ = 4.39, *p* = .015, *η*_*p*_^*2*^ = 0.10). BPD patients showed higher state anger compared to HC and compared to ADHD patients under both conditions (all: *p* ≤ .001). An increase of state anger after stress induction was significant in BPD patients (*p* = .021), but not in HCs and ADHD patients.

### Behavioral aggression

**Fig. 2:**
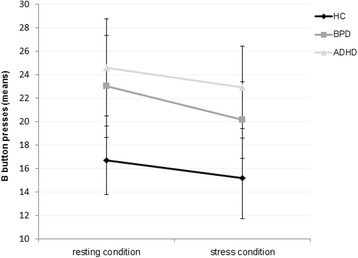
Means with standard errors of behavioural aggression (B button presses in the PSAP) under resting and stress conditions in healthy controls (HC), patients with Borderline Personality Disorder (BPD) and patients with Attention Deficit Hyperactivity Disorder (ADHD)

Means with standard errors of B button presses in the PSAP under resting and stress conditions of all three groups are shown in Fig. 2. The rm-ANCOVA for B button presses revealed no significant effects: main effect of condition (*F*_(1,84)_ =0.99, *p* = .323, *η*_*p*_^*2*^ = 0.01), main effect of group (*F*_(1,84)_ =1.66, *p* = .197, *η*_*p*_^*2*^ = 0.04), and condition x group interaction effect (*F*_(1,84)_ =0.04, *p* = .958, *η*_*p*_^*2*^ < 0.01).

At the end of the whole study participants were asked if they believed they had been playing with a real person. As there have been suggestions that the validity of the PSAP depends on the credibility of the cover story, we also conducted a rm-ANOVA only with those participants who believe the cover story. This sample was composed of 21 HCs, 20 BPD patients and 21 ADHD patients. Similar to the results when analysing the whole sample no significant effects were found: main effect of condition (*F*_(1,59)_ =0.53, *p* = .471, *η*_*p*_^*2*^ = 0.01), main effect of group (*F*_(1,59)_ =0.59, *p* = .557, *η*_*p*_^*2*^ = 0.02), and condition x group interaction effect (*F*_(1,59)_ =0.49, *p* = .615, *η*_*p*_^*2*^ = 0.02). See Additional file 2 for means and standard deviation of PSAP B button presses in the reduced sample.

### Correlation analyses between anger, aggression and emotion regulation capacities

In the BPD sample, a significant negative correlation was found between the DERS score (emotion regulation) and the STAXI total score (anger) (*r* = −0.614, *p* ≤ .001), as well as the BPAQ total score (aggression) (*r* = −0.476, *p* = .009). (Bonferroni correction: α’ = 0.017). There was a trend for a correlations between the DERS total score and the BGLHA in BPD patients (*p* = 0.061). In the ADHD group, the correlation between DERS and BPAQ as well as BGLHA did not reach significance. The correlation with the STAXI scores did not survive Bonferroni correction.

## Discussion

We examined the impact of stress on self-reported and behavioral measures of anger and aggression in female patients with BPD, patients with ADHD and healthy control participants.

The main findings of our study with female participants were: 1) higher self-appraisals of trait anger and aggression in BPD and ADHD patients, 2) higher levels of inwardly directed anger, anger when provoked, general aggression and hostility in BPD patients compared to ADHD patients and 3) a stress-dependence of subjective angry states, but not behavioral aggression, in BPD patients.

### Self-reported trait anger and aggression

Patients reported significantly higher trait anger, anger expression, aggressive and antisocial behavior compared to HCs. These results are consistent with our hypothesis and support previous studies investigating anger and aggression in BPD [18–20, 24, 37, 38] and ADHD patients [32]. Both female patient groups reported to experience more anger, regardless of provocation, compared to HCs. This suggests that lower levels of provocation are needed to evoke subjective anger in BPD and ADHD patients compared to HCs (STAXI “temperament”) and that there is a higher sensitivity towards criticism and rejection in these patients (STAXI “reaction”). This sensitivity to provocation was significantly more pronounced in BPD patients compared to ADHD patients.

Furthermore, both patient groups showed a stronger tendency to suppress feelings of anger, but also to express anger toward other people and/or objects. These are not mutually exclusive ways of anger expression. Whether anger is directed inwardly or outwardly depends on aspects such as the situation, the circumstances or the status of the present persons at the moment of annoyance [35]. Regarding anger expression, individuals may undergo a consecutive process characterized by an initially strong tendency to direct their anger inwardly, until a certain threshold is reached and anger control breaks down, ending up in temper tantrums, throwing objects and/or acting out violently towards others [70]. The intensity of this “belated” externalized anger may be stronger than in cases of immediate outwardly directed anger. In line with the latter findings, BPD and ADHD females rated their anger control capacity lower than HCs. A difference between patients in anger expression was also found in the current study, as female BPD patients displayed a stronger tendency to direct their anger inwardly compared to female ADHD patients. This tendency is probably related to self-destructive behavior (e.g., self-injurious behavior, substance abuse), which is highly prevalent in BPD patients (69–90%; [3, 71, 72]). Research has demonstrated that individuals with BPD are highly sensitive to social rejection [73, 74]. Therefore, even if there is an external origin of annoyance, the tendency to direct their anger mainly inwardly or against themselves may be driven by the fear of abandonment or rejection, if they were to direct their aggression towards another person.

Ratings of aggression in the BPAQ also revealed higher scores in patients concerning general aggression, as well as the components of anger, hostility, verbal and physical aggression. These findings are consistent with previous studies [18, 19, 37]. Moreover, female BPD patients perceived themselves as generally more aggressive and hostile than female ADHD patients. Hostility is an aspect of aggression concerning suspiciousness and the critical appraisal of others and their behavior, which is a prominent interpersonal problem in BPD patients [75, 76]. Furthermore, female and male patients reported that they were more frequently involved in aggressive and antisocial acts (e.g., fighting, assaults) than HCs (BGLHA).

In the present study, BPD patients reported to have more difficulties in emotion regulation compared to HCs and ADHD patients. An elevated self-reported proneness to anger and aggression was significantly associated with deficient emotion regulation capacities in this patient group. Since correlational data do not allowed conclusions about causality, it remains unclear whether enhanced trait anger and aggression impede the acquisition of emotion regulation capacities, or whether deficient emotion regulation skills promote anger experience and aggression. Further studies using for example longitudinal designs (i.e. applying emotion regulation training) are needed for the clarification of this issue.

### Self-reported state anger

Female BPD patients already perceived higher levels of current anger feelings compared to ADHD patients and HCs under resting conditions (STAXI state). After stress induction, female BPD patients reported more anger, whereas no change was observed in ADHD patients and HCs. In male participants, anger feelings also did not change significantly after stress induction. These results suggest that self-perceived anger in female patients with BPD is aggravated by stress.

### Behavioral aggression

While most previous studies found significantly more B button presses in the PSAP in BPD patients [18, 19, 37], female patients in the present study did not make more aggressive responses compared to HCs. After stress induction, we did not observe a stress-dependent change in female patients. One possible explanation for the differing findings might be the presence of a camera in our version of the PSAP, which may have enhanced the self-awareness of the participants. Previous research provides indications for a relationship between higher self-awareness (e.g., presence of a camera) and behaving in a less aggressive manner [77, 78]. There is also evidence suggesting that high emotional awareness enables individuals to behave in an adaptive manner when experiencing negative emotional states [79]. The awareness of one’s current emotional state in our study was possibly enhanced by the questionnaires on tension and anger. Due to the fact that we modified the PSAP our results are not completely comparable to other findings with older versions of the PSAP. For example, we did not find increased levels of behavioral aggression in BPD patients (under baseline conditions) such as New and colleagues [18], McCloskey and colleagues [19] or Dougherty and colleagues [37]. The comparability of the results is further impeded by characteristics of the examined samples. For example, New and colleagues [18] examined BPD patients with comorbid intermittent explosive disorder and Dougherty and colleagues [37] did not exclude bipolar disorder and alcohol abuse. These comorbidities could at least partly influence aggression proneness in BPD.

As one of our objectives was to control for the influence of ADHD symptoms in patients with BPD, we collected a sample of BPD patients without co-morbid ADHD diagnosis. In clinical samples of BPD patients, the presence of comorbid ADHD symptoms is very likely [30, 40] and previous research indicates, that impulse-control problems are more prominent in patients with the combined diagnosis of BPD and ADHD [27, 32, 80]. Thus, the characteristics of our sample may provide an explanation for our results. Future studies should clarify whether there is a difference in the impact of comorbid ADHD on aspects of impulse control in female and male BPD patients.

Our self-report scales may also offer an explanation why there was no elevated proneness to overt behavioral aggression, since the results indicate that our female BPD sample was characterized by a high tendency to internalize their anger. Inwardly direct anger was significantly more pronounced in BPD patients than in ADHD patients. However, we also did not observe an elevated level of behavioral aggression in our female ADHD group. Previous results indicating elevated proneness for aggression in ADHD patients have so far been limited to children and adolescents [5, 7]. Longitudinal studies observing the development of ADHD psychopathology revealed an age-dependent decline of hyperactive and impulsive symptoms [81, 82], which may also implicate a decline of aggressive behavior over time [83].

Interestingly, only male patients in the pilot study reacted more aggressively after stress induction, but no significant changes in aggressive responding were observed in the female samples of the main study (and in healthy males of the pilot study). Previous studies using the PSAP have not revealed differences in the amount of aggressive responses between men and women under conditions without stress induction [18–20]. Whether stress affects aggressive and antisocial behaviour patterns differently in men and women has not been clarified in these studies. There are assumptions that acute stress may in fact enhance prosocial, rather than antisocial behavior, mainly in women (“tend and befriend”; [84]). However, in a recent study by von Dawans and colleagues [85] also healthy male participants showed an improvement in prosocial behavior and unaffected antisocial behavior after stress exposure. Future studies should further clarify the potential differential effects of stress on aggressive behavior in larger samples of BPD and ADHD males.

### General discussion

Strengths of the current study are the moderate sample of well-characterized and unmedicated participants and the comparison of two clinical groups with a healthy control group. In order to differentiate between BPD and ADHD, participants underwent standardized diagnostics, which included structured interviews for BPD (IPDE; [49]) and ADHD (WRAADDS; [59]), beyond self-rating symptom scales, and were conducted by experienced diagnosticians. Furthermore, our BPD patients, ADHD patients and HCs did not differ in age and socioeconomic status. Although there were differences in educational level, no group differences were found in a measurement of intelligence (SPM), therefore, we assumed all three groups had comparable cognitive capacities.

However, some limitations have to be mentioned. It seems important to consider that certain treatments of a sufficient duration could affect symptom severity and thus performance on the task. A special attribute of our study was that all participants were unmedicated (but not all drug-naive) and none of the participants was in inpatient treatment as the investigation took place. Regarding symptom severity, for example the BSL-23 scores indicate that we covered different relative symptom severities in the BPD sample, also including more severely impaired patients (percentile ranks ranged from 14 to 79 in the BPD sample, mean = 51). However, in future studies addressing anger and aggression the treatment history of patients should be assessed in detail.

A critical point might be the type of aggression and the duration of the provocation in the PSAP. Probably penalizing a putative unknown opponent does not represent the type of explosive aggression described in BPD. In BPD patients aggressive behavior in a relational context appears to be of importance as BPD is characterized by chronic interpersonal conflicts [86–88]. Regarding stress induction it should be considered that stress can have different forms. For example stressors which are emphasizing more relational aspects (i.e. Yale InterPersonal Stressor (YIPS); [89]) and induce feelings of exclusion and rejection can also increase self-reported stress and physiological markers such as blood pressure, heart rate and cortisol level [89]. Another approach considers personal/individualized adverse factors such as negative self-descriptions, stressful life-events or trauma-related scripts [90, 91]. Further, as the duration of a stressor seems relevant. As the PSAP performance took 12.5 minutes, there remains the question whether the procedure provokes stress with a lasting effect (for more details see Additional file 3).

As there is evidence for an association between perimenstrual symptomatology and aggressive behavior [92, 93], it is seen as a limitation that we did not control for menstrual cycle, perimenstrual affective symptomatology or hormonal contraception in this study. Further interaction effects between hormonal contraception and stress on prosocial and antisocial behavior are conceivable.

Although we excluded important comorbidities like ADHD, substance abuse and bipolar disorder, we did not exclude further comorbidities such as posttraumatic stress disorder, which is highly prevalent in BPD patients [29, 94], or antisocial personality disorder, which also frequently co-occurs in BPD [29, 95] and ADHD patients [96] and may have an influence on patterns of anger and aggression. Thus, the results of our study have to be interpreted with caution given that there are further comorbidities that could influence the findings. As comorbidity of BPD and ADHD is high, the generalization of our results to clinical samples may be difficult. Future studies should consider adding a clinical sample of patients with BPD and co-morbid ADHD, in order to directly examine possible additive effects of the double diagnosis on anger and aggression. Overall, future research is needed to find differences between BPD and ADHD in order to improve differential diagnosis and prevent treatment malpractice (i.e. putting BPD patients on stimulants).

## Conclusions

Deficits in impulse and anger control can lead to tantrums, assaults or physical fights and can cause severe interpersonal and social problems. Even though aggressive behavior is not necessarily intensified by stress, understanding the effects of stress and interaction with further variables on dysfunctional behavior is important and can help to adjust treatment strategies. In BPD patients, inwardly expressed anger appears to be pronounced. This may be associated with aggressive self-destructive behavior (e.g., self-injury) rather than overt aggressive behavior towards others. Providing functional strategies for anger management seems substantial in the treatment of BPD, even without co-occurring ADHD [97].

## Additional files

Additional file 1: Pilot study. **Table S1.** Demographic and clinical variables in healthy control participants (HC) and patients with Borderline Personality Disorder (BPD) or with Attention Deficit Hyperactivity Disorder (ADHD) taken together. **Table S2.** Ratings of subjective stress and heart rate in resting condition and stress condition in healthy controls (HC) and patients with Borderline Personality Disorder (BPD) or with Attention-Deficit-Hyperactivity-Disorder (ADHD) taken together. **Table S3.** Means and standard deviation of STAXI, BPAQ and BGLHA score results of statistical group comparisons (student’s t-test, *p*-value and effect size) in healthy controls (HC) and patients with Borderline Personality Disorder (BPD) or with Attention-Deficit-Hyperactivity-Disorder (ADHD) taken together. (DOCX 36 kb)
Additional file 2: PSAP subgroup analysis. **Table S4.** Means and standard deviation of PSAP B button presses and statistical group comparison (F-value of ANOVAs, *p*-value and effect size) in healthy controls (HC) and patients with Borderline Personality Disorder (BPD) and with Attention-Deficit-Hyperactivity-Disorder (ADHD) who believed the PSAP cover story. (DOCX 21 kb)
Additional file 3: Lasting effect of stress induction. **Table S5.** Subjective stress ratings under resting condition and after PSAP performance in the stress condition and paired t-tests in HCs, BPD and ADHD patients. (DOCX 17 kb)

## Abbreviations

ADHD: Attention deficit hyperactivity disorder
ADHD-RS: Attention deficit hyperactivity self-rating scale
ANOVA: Analyses of variance
BDI-II: Beck Depression Inventory II
BIS-11: Barratt impulsiveness scale
BGLHA: Brown-Goodwin lifetime history of aggression
BPAQ: Buss Perry aggression questionnaire
BPD: Borderline personality disorder
BSL23: Borderline symptoms list 23
CAARS-S:L: Connor adult ADHD rating scale - self report: long version
CTQ: Childhood trauma-questionnaire
DERS: Difficulties in emotion regulation scale
DES: Dissociative experiences scale
IPDE: International personality disorder examination
MANOVA: Multivariate ANOVA
MDD: Major depressive disorder
OCD: Obsessive compulsive disorder
PFI: Provocation free interval (in the PSAP)
PSAP: Point subtraction aggression paradigm
PTSD: Posttraumatic stress disorder
rm-ANOVA: Repeated measure ANOVA
SCID-I: Structural clinical interview for DSM-IV
STAXI: State-trait anger expression inventory
WRAADDS: Wender-Reimherr adult attention deficit disorder scale
WURS-k: Wender Utah rating scale short version
