# Supplementary material for: Anger and aggression in borderline personality disorder and attention deficit hyperactivity disorder – does stress matter?
Source: Borderline Personal Disord Emot Dysregul. 2017 Mar 17;4:6. doi: 10.1186/s40479-017-0057-5 (PMC5356413; doi:10.1186/s40479-017-0057-5)
Supplement: Additional file 1: — Pilot study. Table S1. Demographic and clinical variables in healthy control participants (HC) and patients with Borderline Personality Disorder (BPD) or with Attention Deficit Hyperactivity Disorder (ADHD) taken together. Table S2. Ratings of subjective stress and heart rate in resting condition and stress condition in healthy controls (HC) and patients with Borderline Personality Disorder (BPD) or with Attention-Deficit-Hyperactivity-Disorder (ADHD) taken together. Table S3. Means and standard deviation of STAXI, BPAQ and BGLHA score results of statistical group comparisons (student’s t-test, p-value and effect size) in healthy controls (HC) and patients with Borderline Personality Disorder (BPD) or with Attention-Deficit-Hyperactivity-Disorder (ADHD) taken together. (DOCX 36 kb) [file 40479_2017_57_MOESM1_ESM.docx]

**Supplementary material 1: Pilot study**

Because of the modifications we made in the PSAP, we first conducted a pilot study with male BPD and ADHD patients and HCs to test whether our version of the PSAP was sensitive for stress-dependent changes in behavioral aggression.

**Methods**

Procedure, assessments and inclusion/exclusion criteria were identical in the pilot and main study.

Sample

Our sample consisted of 40 males between 22 and 53 years of age, which were recruited in the Department of Psychosomatic Medicine and Psychotherapy and the Department of Psychiatry and Psychotherapy at the Central Institute of Mental Health (CIMH) in Mannheim, Germany. No participants were excluded from data analyses, thus the final sample consisted of 20 male patients with either BPD or ADHD (each 10) and 20 HCs.

**Results**

The means and SD for demographic and clinical variables, as well as patients’ comorbid psychiatric disorders are presented in supplementary Table S1.

**Table S1.** Demographic and clinical variables in healthy control participants (HC) and patients with Borderline Personality Disorder (BPD) or with Attention Deficit Hyperactivity Disorder (ADHD) taken together

|  | **HC (*n*= 20)**  M ± S.D. | **Patients (*n*= 20)**  M ± S.D. | ***T /*** ***χ²*** | ***p*** | ***d*** |
| --- | --- | --- | --- | --- | --- |
| **Age** | 31.60 ± 5.48 | 35.05 ± 9.14 | -1.45 | .158 |  |
| **Intelligence (IQ)**  Raven SPM | 112.15 ± 10.50 | 105,55 ± 11.12 | 1.93 | .061 |  |
| **Income,** *n (%)*  100-300€  350-500€  550-700€  750-1000€  + 1000€ | 0 (0)  2 (10)  0 (0)  3 (15)  15 (75) | 1 (5)  4 (20)  3 (15)  4 (20)  8 (40) | 6.94 | .139 |  |
| **Years of education,** *n (%)*  Less than 9 years  9 years  10 years  13 years | 0 (0)  0 (0)  4 (20)  16 (80) | 1 (5)  0 (0)  5 (25)  14 (70) | 1.24 | .537 |  |
| **Current co-morbidities,** *n (%)*  MDD  Anxiety disorder  Substance abuse  Eating disorder  PTSD |  | 3 (15)  4 (20)  0 (0)  4 (20)  4 (20) |  | |  |
| **WURS-k**^#^ | 13.00 ± 12.12 | 43.15 ± 8.98 | -8.94 | ≤.001 | 2.83 |
| **ADHD-SB** | 7.60 ± 4.85 | 29.10 ± 6.34 | -12.05 | ≤.001 | 3.81 |
| **CAARS** | 39.40 ± 21.48 | 112.65 ± 16.30 | -12.15 | ≤.001 | 3.84 |
| **BDI-II** | 4.20 ± 6.15 | 25.65 ± 16.83 | -5.35 | ≤.001 | 1.69 |
| **BSL23** | 2.55 ± 4.40 | 35.15 ± 24.32 | -5.94 | ≤.001 | 1.87 |

Data are presented in means ± standard deviations, statistical group comparisons by student´s *t*- test and ***χ²*** -test for income (*df*=4) and education (*df*=2); *p*-value; effect size in *d****;***

MDD= Major Depressive Disorder; PTSD= Posttraumatic stress disorder; OCD= Obsessive Compulsive Disorder; WURS-k= Wender Utah Rating scale short version; ADHD-SB= Attention Deficit Hyperactivity self-rating; CAARS= Connor Adult ADHD Rating Scale; BDI-II = Beck Depression Inventory II; BSL-23= Borderline Symptom List-23

The means and SD for subjective stress ratings and heart rate are depicted in the supplementary Table S2. The rm-ANOVAs with heart rate as dependent variable revealed a significant main effect of condition (*F*_(1,36)_=49.23, *p*≤.001, *η_p_²*=0.58), Also, in the rm-ANOVA for subjective rating a significant main effect of condition was found (*F*_(1,38)_=14.72, *p*≤.001, *η_p_²*=0.28) and a significant effect of group (*F*_(1,38)_=6.17, *p*≤.05, *η_p_²*=0.14) with higher stress levels in the patients group. Significantly higher subjective stress ratings and increased heart rate after MMST performance in both groups (*p*≤.05-.001) indicate successful stress manipulation.

**Table S2:** Ratings of subjective stress and heart rate in resting condition and stress condition in healthy controls (HC) and patients with Borderline Personality Disorder (BPD) or with Attention-Deficit-Hyperactivity-Disorder (ADHD) taken together

|  | **Stress ratings**  M ± S.D. | | **paired *t*-tests** | **Heart rate**  M ± S.D. | | **paired *t*-tests** |
| --- | --- | --- | --- | --- | --- | --- |
|  | **resting**  **condition** | **stress**  **condition** |  | **resting**  **condition** | **stress**  **condition** |  |
| **HC**^#^  **(*n*= 20)** | 2.65 ± 1.53 | 4.00 ± 1.81 | *t*_(19)_= -3.50  *p*≤ .01  *d*= .81 | 76.89 ± 8.33 | 96.89 ± 18.18 | *t*_(17)_= -4.84  *p*≤ .001  *d*=1.41 |
| **Patients**  **(*n*= 20)** | 4.15 ± 2.11 | 5.15 ± 2.25 | *t*_(19)_= -2.10  *p*≤ .05  *d*=.46 | 78.77 ± 12.64 | 91.45 ± 10.81 | *t*_(19)_= -5.27  *p*≤ .001  *d*=1.08 |
| ***t*-tests** | *t*_(38)_= -2.57  *p*≤ .05  *d*= .81 | *t*_(38)_= -1.78  *p*= .083  - |  | *t*_(36)_= -0.54  *p*= .595  - | *t*_(27)_= 1.11  *p*= .279  - |  |

^#^ = heart rate data of two HC are missing due to technical problems (*n*= 18)

Although we had no specific hypothesis concerning self-reported anger and aggression in the pilot study, for reasons of completeness, we report data from the STAXI, BPAQ and BGLHA, as well as the STAXI state scores under the resting and stress conditions in the supplementary Table S3.

The means with SD and statistics of B button presses in the PSAP under the resting and stress conditions of both groups are also shown in the supporting Table S3. The rm-ANOVA revealed a significant Group x Condition interaction effect (*F*_(1,38)_= 4.39, *p*≤.05, *η_p_²*=0.10). There was no significant main effect for group or condition. After stress induction patients, but not HCs, made significantly more B button presses compared to the resting condition (*p*=.033).

**Table S3.** Means and standard deviation of STAXI, BPAQ and BGLHA score results of statistical group comparisons (student´s t-test, p-value and effect size) in healthy controls (HC) and patients with Borderline Personality Disorder (BPD) or with Attention-Deficit-Hyperactivity-Disorder (ADHD) taken together

|  | **HC (*n*= 20)**  M ± S.D. | **Patients (*n*= 20)**  M ± S.D. | ***t*** | ***p*** | | ***d*** |
| --- | --- | --- | --- | --- | --- | --- |
| **Trait measures** |  |  |  | | |  |
| **STAXI**  total  temperament  reaction  anger in  anger out  anger control | 15.40 ± 3.72  6.60 ± 1.54  8.80 ± 2.57  14.70 ± 4.94  10.45 ± 2.35  27.10 ± 3.91 | 25.50 ± 5.83  12.20 ± 3.62  13.30 ± 2.90  21.30 ± 5.96  15.85 ± 3.51  20.70 ± 3.08 | -6.54  -6.37  -5.19  -3.81  -5.71  5.76 | ≤.001  ≤.001  ≤.001  ≤.001  ≤.001  ≤.001 | | 2.07  2.01  1.641.211.811.82 |
| **BPAQ**  total  anger  physical  verbal  hostility | 46.90 ± 8.49  10.45 ± 2.89  13.35 ± 3.68  9.90 ± 1.55  13.20 ± 4.14 | 69.35 ± 14.92  18.85 ± 3.83  17.60 ± 6.00  13.20 ± 2.71  19.70 ± 6.64 | -5.85  -7.83  -2.70  -4.73  -3.72 | ≤.001  ≤.001  ≤.05  ≤.001 ≤.001 | | 1.852.480.851.491.17 |
| **BGLHA^#^**  total | 0.95 ± 1.43 | 9.94 ± 6.58 | -5.52 | | ≤.001 | 1.89 |
| **State measures** |  |  |  | | |  |
| **STAXI state**  resting  stress | 10.63 ± 1.11  10.80 ± 1.28 | 13.60 ± 5.09  14.40 ± 5.52 | -2.55  -2.84 | | ≤.05  ≤.05 | 0.81  0.90 |
| **PSAP B button presses**  resting  stress | 25.65 ± 21.80  19.20 ± 20.40 | 17.50 ± 12.21  25.45 ± 22.41 | 1.46  -0.92 | | .153  .362 | 0.460.29 |

STAXI= State-Trait Anger Expression Inventory; BPAQ= Buss Perry Aggression Questionnaire; BGLHA= Brown-Goodwin Lifetime History of Aggression; PSAP= Point Subtraction Aggression Paradigm

^#^ BGLHA: smaller sample size due to missing values: BPD (*n*=17)

**Discussion**

This modified version of the PSAP was sensitive for stress-dependent changes in behavioral aggression. In male patients we found a stress-related increase in aggressive responses, but no group difference independent of stress. In the next step we applied this version of the PSAP in a larger sample of BPD, ADHD and healthy females (see main study, also for further discussion).
