# Supplementary material for: Anger and aggression in borderline personality disorder and attention deficit hyperactivity disorder – does stress matter?
Source: Borderline Personal Disord Emot Dysregul. 2017 Mar 17;4:6. doi: 10.1186/s40479-017-0057-5 (PMC5356413; doi:10.1186/s40479-017-0057-5)
Supplement: Additional file 2: — PSAP subgroup analysis. Table S4. Means and standard deviation of PSAP B button presses and statistical group comparison (F-value of ANOVAs, p-value and effect size) in healthy controls (HC) and patients with Borderline Personality Disorder (BPD) and with Attention-Deficit-Hyperactivity-Disorder (ADHD) who believed the PSAP cover story. (DOCX 21 kb) [file 40479_2017_57_MOESM2_ESM.docx]

**Supplementary analysis 2: PSAP subgroup analysis**

As there have been suggestions that the validity of the PSAP depends on the credibility of the cover story, we also conducted a rm-ANOVA only with those participants who believe the cover story. This sample was composed of 21 HCs, 20 BPD patients and 21 ADHD patients. Similar to the results when analysing the whole sample no significant effects were found: main effect of condition (*F*_(1,59)_ =0.53, *p*=.471, *η_p_²*=0.01), main effect of group (*F*_(1,59)_ =0.59, *p*=.557, *η_p_²*=0.02), and condition x group interaction effect (*F*_(1,59)_ =0.49, *p*=.615, *η_p_²*=0.02). See Table S4 for means and standard deviation of PSAP B button presses in the reduced sample.

**Table S4.** Means and standard deviation of PSAP B button presses and statistical group comparison (F-value of ANOVAs, p-value and effect size) in healthy controls (HC) and patients with Borderline Personality Disorder (BPD) and with Attention-Deficit-Hyperactivity-Disorder (ADHD) who believed the PSAP cover story

|  | **HC (*n*= 21)**  M ± S.D. | **BPD (*n*= 20)**  M ± S.D. | **ADHD (*n*= 21)**  M ± S.D. | ***F*** | ***p*** | ***η_p_²*** |
| --- | --- | --- | --- | --- | --- | --- |
| **PSAP B button presses**  resting  stress | 18.29 ± 10.44  19.67 ± 20.21 | 27.00 ± 26.19  22.30 ± 15.94 | 23.90 ± 22.49  21.76 ± 19.75 | 0.94  0.11 | .398  .893 | .03  <.01 |

PSAP= Point Subtraction Aggression Paradigm
