# Supplementary material for: Anger and aggression in borderline personality disorder and attention deficit hyperactivity disorder – does stress matter?
Source: Borderline Personal Disord Emot Dysregul. 2017 Mar 17;4:6. doi: 10.1186/s40479-017-0057-5 (PMC5356413; doi:10.1186/s40479-017-0057-5)
Supplement: Additional file 3: — Lasting effect of stress induction. Table S5. Subjective stress ratings under resting condition and after PSAP performance in the stress condition and paired t-tests in HCs, BPD and ADHD patients. (DOCX 17 kb) [file 40479_2017_57_MOESM3_ESM.docx]

**Supplementary material 3**

**Lasting effect of stress induction**

In order to know whether the stress induced by MMST lasts till the end of the PSAP performance, we examined the stress change from stress induction till the end of the PSAP. (A stress rating was also assessed after the PSAP performance.) Stress ratings in all three groups declined significantly (p < .05) at the end of the PSAP. We therefore also compared these ratings at the end of the PSAP with the ratings at resting condition (see Table 1).

**Table S5**. Subjective stress ratings under resting condition and after PSAP performance in the stress condition and paired t-tests in HCs, BPD and ADHD patients

|  | Stress rating | | paired t-tests |
| --- | --- | --- | --- |
|  | Resting condition | Stress condition after PSAP performance |  |
| HC | 1.63 ± 1.47 | 2.77 ± 2.19 | *t*_(29)_= -4.07  *p*≤ .001  *d*= 0.61 |
| BPD | 3.76 ± 1.70 | 4.66 ± 2.09 | *t*_(28)_= -1.90  *p*= .068  *d*= 0.47 |
| ADHD | 3.50 ± 2.25 | 4.86 ± 2.49 | *t*_(27)_= -2.50  *p*= .019  *d*= 0.57 |

Although the stress ratings decrease until the end of the PSAP, the ratings still differ significantly from the ratings under resting conditions (expect in the BPD group with only a trend).
